# Prognostic Alternative mRNA Splicing in Adrenocortical Carcinoma

**DOI:** 10.3389/fendo.2021.538364

**Published:** 2021-03-12

**Authors:** Weiwei Liang, Fangfang Sun

**Affiliations:** ^1^ Department of Endocrinology, The Second Affiliated Hospital, Zhejiang University School of Medicine, Hangzhou, China; ^2^ Key Laboratory of Cancer Prevention and Intervention, China National Ministry of Education, The Second Affiliated Hospital, Cancer Institute, Zhejiang University School of Medicine, Hangzhou, China

**Keywords:** alternative splicing, adrenocortical carcinoma, AUC, bioinformatics, prognostic model

## Abstract

**Background:**

This paper aims to identify alternative RNA splicing landscape and its prognostic value in adrenocortical carcinoma.

**Methods:**

The alternative splicing events data with corresponding clinical information data of 79 ACC patients were obtained from the Cancer Genome Atlas and SpliceSeq package. Prognosis-associated AS events by using univariate Cox regression analysis were selected. Gene functional enrichment analysis demonstrated the potential pathways enriched by survival-associated AS. Prognosis-related splicing events were submitted to develop moderate predictors using Lasso regression model.

**Results:**

One thousand five survival-associated alternative splicing events were identified. The prognostic genes included *ATXN2L, MEIS1*, *IKBKB*, *COX4I1*. Functional enrichment analysis suggested that prognostic splicing events are associated with Wnt signaling pathway. A prediction model including 12 alternative splicing events was constructed by Lasso regression using train set. ROC analysis showed good performance of the prediction model in test set. Then, a nomogram integrating the clinical-pathological factors and riskscore was constructed for predicting 1‐ and 3‐year survival rate.

**Conclusion:**

Our data provide a comprehensive bioinformatics analysis of AS events in ACC, providing biomarkers for disease progression and a potentially rich source of novel therapeutic targets.

## Introduction

Cancers are often associated with aberrant proteins. Cancer genome research showed the number of protein-coding genes was limited, which makes it difficult to account for the diverse proteomic phenotypes. Alternative RNA splicing is a key step of post-transcriptional gene expression regulation (1). There were seven types of alternative splicing events: Exon Skip (ES), Mutually Exclusive Exons (ME), Retained Intron (RI), Alternate Promoter (AP), Alternate Terminator (AT), Alternate Donor site (AD), and Alternate Acceptor site (AA). Through alternative processing, genes can produce distinct RNA isoforms. Alternative splicing contributes to the protein diversity for 90% of human gene expression. Thus, it is not surprising that defects in alternative splicing are involved in tumorigenesis (2). AS also provided targets of cancer treatment (3). Characterization of the AS landscape provides a wealth of insight into cancers with excellent prognostic value.

Our study aimed to detect AS landscape in adrenocortical carcinoma (ACC). ACC is a rare endocrine tumor with generally poor prognosis. Study showed median overall survival (OS) is 3.21 years, and 5-years overall survival of ACC is below 40% in most series (4–9). ACC counts 0.2% of all cancer deaths in the United States, while the incidence of ACC is only 0.72 per million cases per year (10). The prognosis of ACC is very heterogeneous. Identifying prognostic factors of ACC is significant for clinical decision making. The recent studies had revealed some prognosis-associated molecular mechanisms of ACC (11). However, studies of alternative splicing in ACC are still lacking.

To unravel AS landscape and its prognostic value in ACC, this study utilized the rich data from The Cancer Genome Atlas (TCGA) consortium. We systematically profiled the alternative splicing events in ACC cohort from TCGA. Survival-associated alternative splicing events were identified. Then, we constructed prognostic model for ACC patients.

## Methods

### Data Sources

The RNA sequencing data with corresponding clinical information data of 79 ACC patients were obtained from the Cancer Genome Atlas (https://tcga-data.nci.nih.gov/tcga/) and SpliceSeq package (12, 13). Clinical information data included age, gender, TNM stage, overall survival (OS), and live/dead status. Totally seven types of alternative splicing events were all involved in our study.

### Survival Analysis

**Figure 1:**
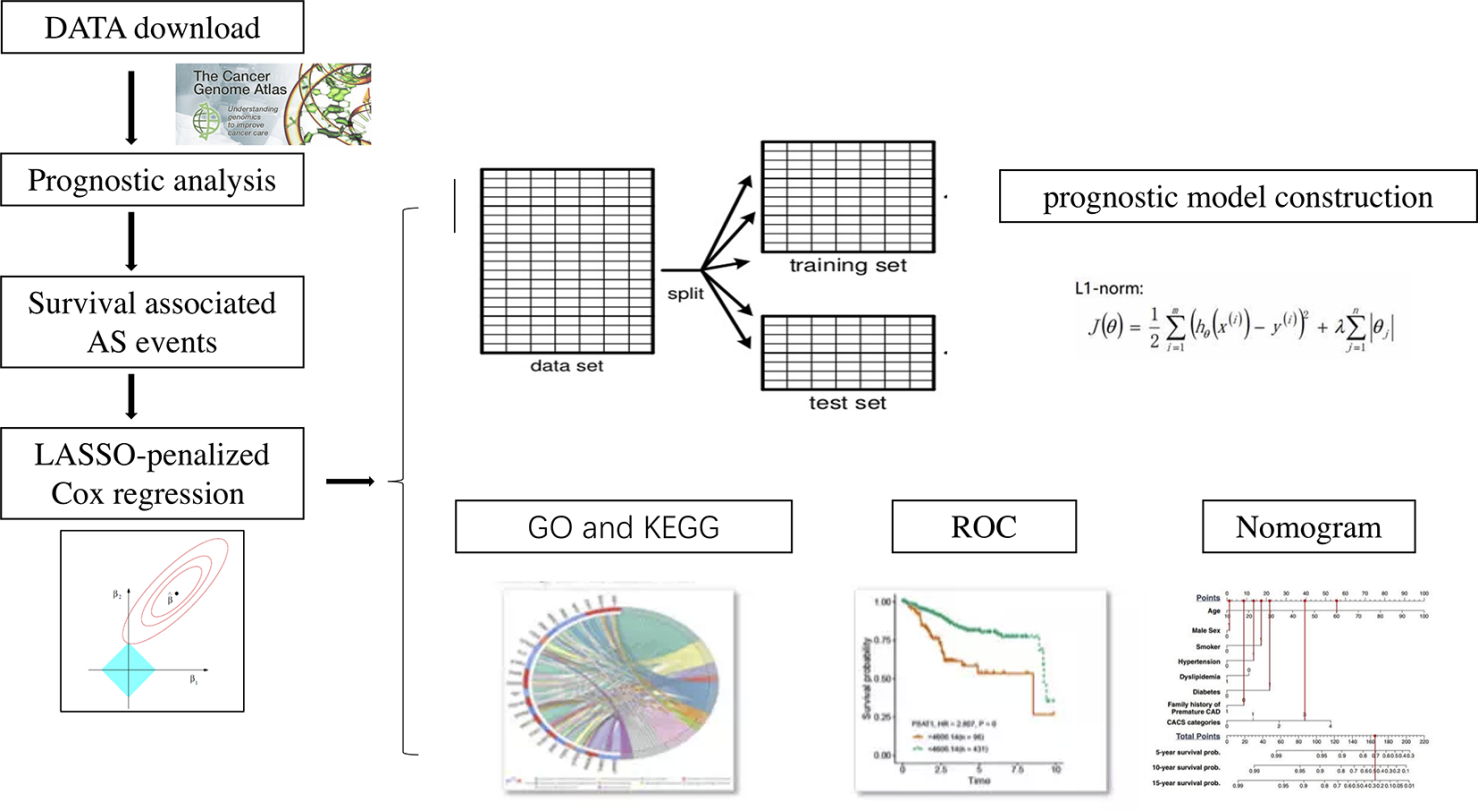
The flow chart for the experimental design.

The flow chart for the experimental design is displayed in **Figure 1**. Univariate Cox regression was first used to access the association between all alternative splicing events and OS. Multiple testing correction was done using Bonferroni adjustment. P. adj < 0.05 was set as the cut-off criterion. Data set was divided into training set and texting set. A least absolute shrinkage and selection operator (LASSO) penalized Cox regression model was used to select the most useful alternative splicing events as prognostic predictors. A prognostic model was then constructed based on the percent-spliced-in (PSI) level of the selected alternative splicing events using Cox regression coefficients. The patients were classified into low‐risk group and high‐risk group according to the median value of the risk scores. The area under the curve (AUC) of the receiver-operator characteristic (ROC) curve for prognostic model was generated. The survival outcome endpoint was selected as 1 year and 3 years because fewer events occurred after 3 years in survival analyses. All reported p values were two-sided. p < 0.05 was set as the cut-off criterion.

All analyses were performed using Bioconductor in R (version 3.5.3). Upset plot was done using the “UpsetR” package (14). Univariate Cox regression was done using the “survival” package (15). The LASSO analysis was undertaken using the “glmnet” package (16). ROC curve was performed with the function of “survivalROC” (17).

### Functional Analysis

We used Gene ontology analysis (GO) to identify characteristic biological attributes of the genes with survival-associated AS events. We performed Kyoto Encyclopedia of Genes and Genomes pathway (KEGG) (18) enrichment analysis to identify functional attributes. GO and KEGG analysis was done using the “DOSE” (19), “org.Hs.eg.db” (20), “clusterProfiler” (21), and “pathview” package (22) in R. For the visualization of the data, the “ggplot2” package (23) was used.

### Construction of the Nomogram

Nomogram were generated using both the riskscore value and clinicopathological factors for predicting patient survival probability at 1 and 3 years. Nomogram provide a quantitative and intuitive method to assess the association between variables and survival. Each value within these variables was assigned a score on the point scale. Locate the sum on the Total Points scale and vertically project it onto the bottom axis, we were easily determined the estimated probability of the 1- and 3-year survival probability. Nomograms plots were done with the “rms” package (24) and the “survival” package (15).

## Results

### AS Events Profiles in TCGA ACC Cohort

RNA-seq files and clinical information of 79 ACC patients from TCGA database were downloaded. Two patients without complete follow-up information or complete clinical information were excluded. A total of 34,419 mRNA splicing events in 8,993 genes were detected, which contains 124 MEs in 122 genes, 2,395 RIs in 1,605 genes, 2,707 AAs in 1,960 genes, 2,382 ADs in 1,688 genes, 6,341 APs in 2,575 genes, 8,201 ATs in 3,575 genes, 12,269 ESs in 5,336 genes. These results indicated one gene has several types of splicing events. Among them, ES events were the most common type.

### Identify Survival-Associated AS Events

**Figure 2:**
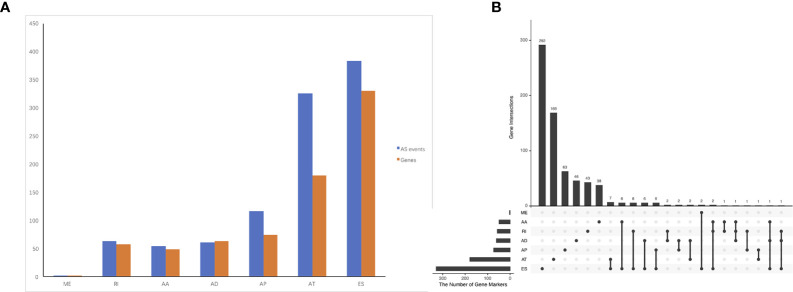
Prognosis-related alternative splicing (AS) events in ACC. **(A)** numbers of identified prognosis-associated AS events and genes. **(B)** UpSet plot showed the interactions among the seven types of prognosis-associated AS events. ES was the predominant event. One gene may have up to four types of AS events.

The association of the alternative splicing events with overall survival was studied by univariate survival analyses. Multiple testing correction was done using Bonferroni adjustment. A total of 1,005 survival-associated alternative splicing events (p.adj < 0.05) were detected. Two ME events in 2 genes, 63 RI events in 58 genes, 54 AA events in 49 genes, 61 AD events in 63 genes, 117 AP events in 74 genes, 325 AT events in 180 genes, and 383 ES events in 330 genes were identified as prognosis-associated AS events (**Figure 2A**). UpSet plot (**Figure 2B**) vividly revealed that ES was the predominant event. There were four genes that have three prognosis-related events (*ATXN2L*, *MEIS1*, *IKBKB*, *COX4I1*).

### Molecular Characteristics of Genes With Survival-Associated AS Events

**Figure 3:**
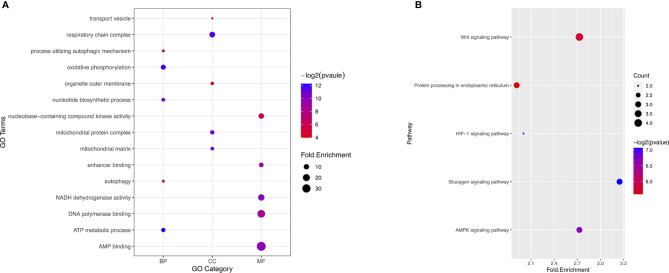
GO and KEGG analysis of the genes with prognosis-associated AS events. **(A)** GO analysis. X axis represents three types of GO. The node size is representative of fold.enrichment level, and the color represents −log2(pvalue). MF, molecular function; CC, cellular component; BP, biological process. **(B)** KEGG analysis. X axis represents fold.enrichment. The node size is representative of gene counts. The color represents −log2(pvalue).

To reveal the molecular characteristics of genes with survival-associated AS events, GO and KEGG analyses were conducted (**Figure 3**). KEGG analysis showed metabolic pathways was the most significant enriched pathway for genes with survival-associated AS events was Wnt signaling pathway (**Figure 3B**).

### Prognostic Predictors for ACC Patients

**Figure 4:**
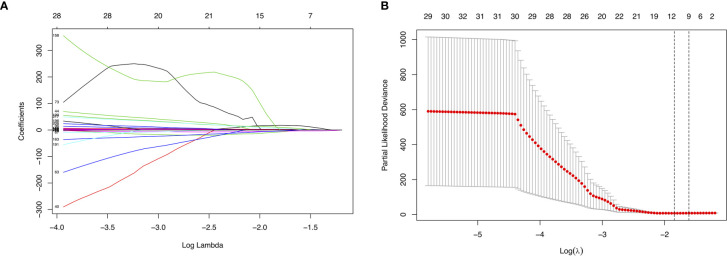
Parameter selection in LASSO analysis. **(A)** The relationship between the coefficient and the Log(Lambda). The numbers above the graph represent the number of AS involved in the LASSO model. **(B)** Tuning parameter selection in the LASSO model. The partial likelihood deviance is plotted against Log(Lambda), where Lambda is the tuning parameter. Partial likelihood deviance values are shown, with error bars representing s.e. The dotted vertical lines are drawn at the optimal values by minimum criteria and 1- s.e. criteria.

We randomly divided the data set into a training set and an internal validation set according to a 17:3 ratio. First, LASSO analysis was undertaken to identify key prognostic marker to develop prognostic model in alternative splicing seven types (**Figure 4A**). Twelve alternative splicing events were retained according to the optimal lambda value (**Figure 4B**, optimal *Lambda*.min = 0.2187).

**Figure 5:**
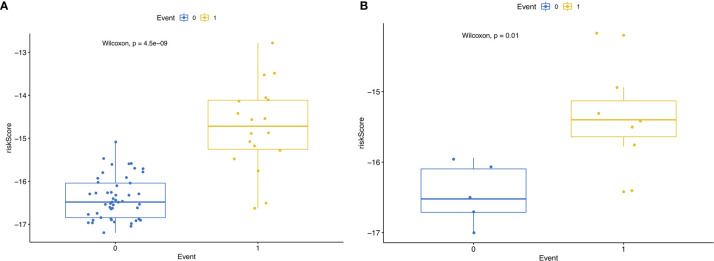
**(A)** RisksRiskScore of patients with different survival status in train set. 0 representing death and 1 representing alive. **(B)** RisksRiskScore of patients with different survival status in test set. 0 representing death and 1 representing alive.

**Figure 6:**
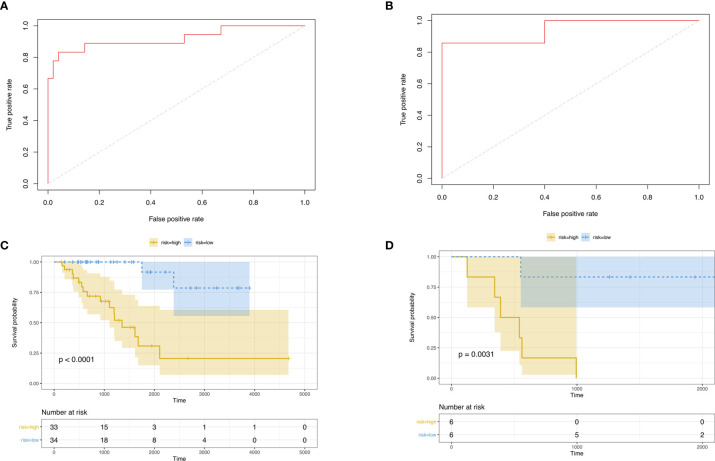
**(A)** ROC curve of prognostic predictors for ACC in test set. **(B)** ROC curve of prognostic predictors for ACC in test set. **(C)** The KM survival analysis of highand low-risk group in train set. Yellow curve, high-risk patient group; blue curve, low-risk patient group. **(D)** The KM survival analysis of high- and low-risk group in test set. Yellow curve, high-risk patient group; blue curve, low-risk patient group.

Then, we built the final prognostic predictors including the independent prognostic AS events. The formula for the riskscore can be found in **Supplementary Table 1**. The riskscore for each patient was showed in **Supplementary Tables 2** and **3**. The patients were classified into low‐risk group and high‐risk group according to the median value of the risk scores. **Figure 5** showed patients in high-risk group had poor survival. Then, we applied ROC curves to compare the efficiency of prognostic models. Result showed the prognostic model had a good performance in distinguishing good or poor survival in patients (**Figures 6A, B**, AUC = 0.92 and 0.94 in train set and test set, respectively). The KM analysis showed significant difference of survival between high- and low-risk group in both train set and test set (**Figures 6C, D**).

### Construction of a Nomogram for Predicting 1‐ and 3‐Year Survival

**Figure 7:**
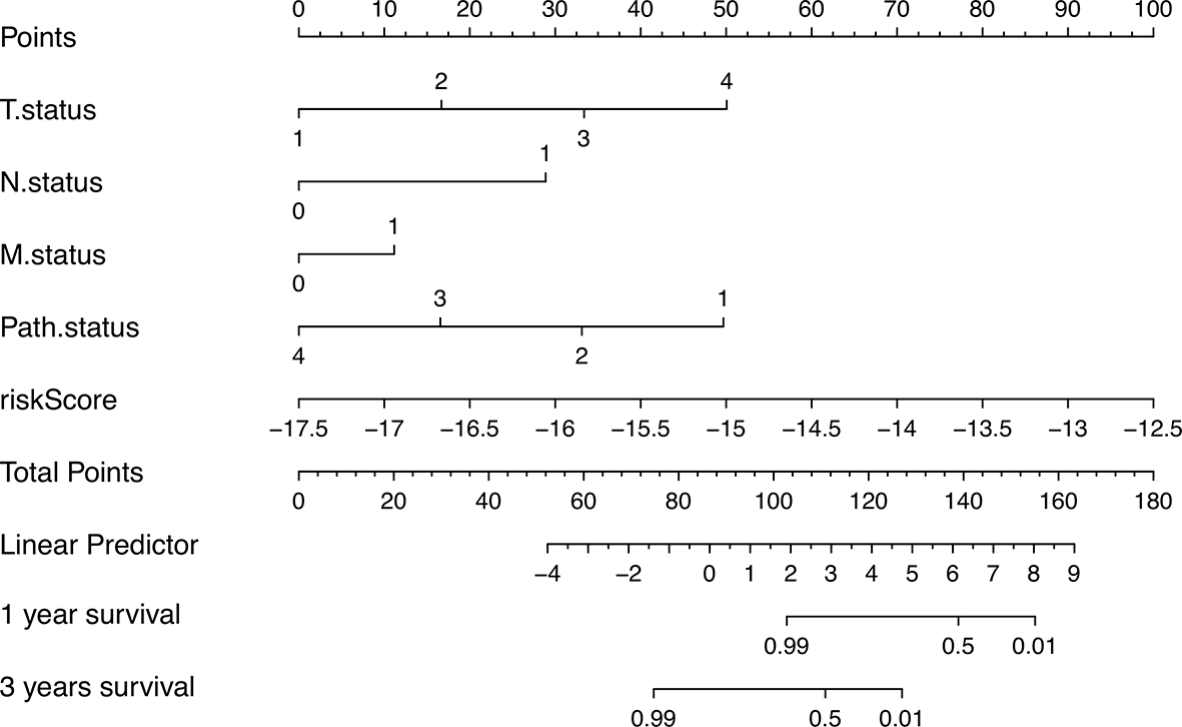
The nomogram for predicting 1‐ and 3‐year survival.

In order to better apply the risk signature, we collected 77 ACC patients with detailed clinical information including T status, N status, M status, TNM stage, and gender. Subsequently, a nomogram integrating the five factors and riskscore was constructed for predicting 1‐ and 3‐year survival rate (**Figure 7**). In the nomogram, the patients’ 1‐ and 3‐year survival rates were estimated by the total points obtained by adding up the point of each factor.

## Discussion

Alternative splicing is an important biological process. Protein-coding genes were limited, it is alternative splicing that provides the potential to generate diversity at RNA and protein levels. Alternative splicing changes are frequently observed in cancer. For example, Goldstein’s study showed an alternative splicing change in *NFE2L2* leads to the loss of a protein domain and the interaction with its negative regulator *KEAP1*, thereby providing an alternative mechanism for the activation of an oncogenic pathway. There were studies showing alternative splicing closely associated with cancer patients’ survival. Survival-associated alternative splicing had been identified in other types of cancer like lung cancer, thyroid cancer.

To our knowledge, it is the first paper focusing on survival-associated AS in ACC. In this study, we systematically analyzed the survival-associated AS events in 79 ACC patients from TCGA. Survival-associated AS events were identified. We built well performed prognostic predictors.

In our study, we identified several prognostic genes and potential molecular function. The prognostic genes included *ATXN2L*, *MEIS1*, *IKBKB*, *COX4I1*. Studies showed these prognostic genes played critical roles in cancer biology. For example, Lin’s study showed *ATXN2L* upregulated by epidermal growth factor promotes gastric cancer cell invasiveness (25). Deregulation of *MEIS1* had been reported in cancers like prostate cancer (26), ovarian cancer (27), lung cancer (28). *MEIS1* transcriptionally regulated the expression of hypoxic tumor markers, namely Hif-1α and Hif-2α, which had crucial roles in tumorigenesis (29). Meysam’s study showed Msi1 is an important activator for Wnt pathways in esophageal squamous cell carcinoma progression and metastasis (30). *COX4I1* is a nuclear gene that encodes the common isoform of cytochrome c oxidase (COX) subunit 4 (COX 4-1), which is an integral regulatory part of the mitochondrial respiratory chain (31). COX4 was a biomarker for breast cancer (32) and medullary thyroid cancer (33). *IKKBK*, a key molecule in signaling to the transcription factor NFκB (34), is a very important regulator in tumorigenesis (35–39). Further investigation is necessary to clarify underlying biological links between these genes and ACC. Further investigation is necessary to clarify underlying biological links between these genes and ACC.

Adrenocortical carcinoma (ACC) is an endocrine malignancy with dismal prognosis. Current knowledge of predictors of survival in adrenocortical carcinoma was limited to clinical parameters like increasing age, higher comorbidity index, grade, and stage of ACC (40). Studies of molecular prognostic markers of ACC are limited. Lacombe et al. reported sterol-O-acyl transferase 1 as a prognostic marker (41). A simple prognostic marker based on the differential expression of two genes (BUB1B-PINK1) was developed by de Reyniès (42). Three studies investigated DNA methylation profiles in ACC and showed that a subset of ACC presents hypermethylation of CpG islands (43–45).

Genomics, transcriptomics, proteomics, metabolomics, and other omics technologies have been identified as key technologies to find answers to unresolved clinical questions. There were many studies attempting to dig meaningful information from the large volume data. Predictive model is a good entry point for basic medicine to clinical medicine. One problem of the traditional variable selecting methods such as cox regression and logistic regression is the over-fitting when facing omics data (46). LASSO regression is a good solution of this problem (47). In our study, we constructed prognostic model with 12 predictors using Lasso regression. The AUC of the prognostic predictors was 0.89. Our prognostic predictors are promising in providing new perspective to understand ACC heterogeneity and to develop new ACC prognostication strategies.

There was some limitation of this study. Functional studies that further delineate the biologic basis of the impact of dysregulated AS events in ACC are needed. Another large independent validation cohort should be used to validate our result in the future.

In conclusion, our data provide a comprehensive bioinformatics analysis of AS events in ACC. A well performed prognostic predictors was constructed for risk stratification in ACC. Our study provided biomarkers for disease progression and a potentially rich source of novel therapeutic targets. We believe these findings could contribute to clinical cancer management.

## Data Availability Statement

Publicly available datasets were analyzed in this study. The RNA sequencing expression data and clinical information data of 79 ACC patients were obtained from TCGA data portal (https://tcga-data.nci.nih.gov/tcga/).

## Author Contributions

WL analyzed and interpreted the data and was the major contributor in writing the manuscript. FS formatted the figures and tables. All authors contributed to the article and approved the submitted version.

## Funding

This paper was funded by Zhejiang Provincial Natural Science Foundation of China [grant number LQ20H160021].

## Conflict of Interest

The authors declare that the research was conducted in the absence of any commercial or financial relationships that could be construed as a potential conflict of interest.
