# Supplementary material for: Prognostic Alternative mRNA Splicing in Adrenocortical Carcinoma
Source: Front Endocrinol (Lausanne). 2021 Mar 12;12:538364. doi: 10.3389/fendo.2021.538364 (PMC7994755; doi:10.3389/fendo.2021.538364)
Supplement: Supplementary file 1 [file DataSheet_1.docx]

$$RiskScore=\sum PSI level of splicing*coresponding Coef level$$

| Splicing | Coef |
| --- | --- |
| ID_46443 | 2.40E+00 |
| ID_76157 | -8.56E+00 |
| ID_24580 | 2.48E+00 |
| ID_7868 | -4.58E+00 |
| ID_13671 | 1.78E+01 |
| ID_48789 | 3.67E-01 |
| ID_55743 | 3.64E-01 |
| ID_35855 | 1.86E+00 |
| ID_63470 | 1.25E+01 |
| ID_16730 | -4.34E+00 |
| ID_60188 | 1.09E+01 |
| ID_62215 | 7.55E+00 |

Sup. Table 1. Formula for the RiskScore. Coef level of each splicing.

|  | OS | Event | riskScore | risk |
| --- | --- | --- | --- | --- |
| TCGA.OR.A5J1 | 3.71 | 1 | -15.18 | high |
| TCGA.OR.A5J2 | 4.59 | 1 | -15.76 | high |
| TCGA.OR.A5J5 | 1.00 | 1 | -13.49 | high |
| TCGA.OR.A5J7 | 1.34 | 1 | -14.14 | high |
| TCGA.OR.A5J8 | 1.59 | 1 | -14.11 | high |
| TCGA.OR.A5J9 | 3.24 | 0 | -16.32 | low |
| TCGA.OR.A5JA | 2.53 | 1 | -14.57 | high |
| TCGA.OR.A5JB | 1.51 | 1 | -14.05 | high |
| TCGA.OR.A5JC | 4.79 | 1 | -16.51 | low |
| TCGA.OR.A5JE | 5.77 | 1 | -15.48 | high |
| TCGA.OR.A5JJ | 0.85 | 0 | -15.59 | high |
| TCGA.OR.A5JK | 3.44 | 0 | -16.29 | low |
| TCGA.OR.A5JL | 1.84 | 0 | -16.92 | low |
| TCGA.OR.A5JO | 2.44 | 0 | -16.47 | low |
| TCGA.OR.A5JP | 0.41 | 0 | -15.78 | high |
| TCGA.OR.A5JQ | 1.85 | 0 | -16.84 | low |
| TCGA.OR.A5JR | 10.10 | 0 | -16.62 | low |
| TCGA.OR.A5JS | 1.05 | 0 | -15.08 | high |
| TCGA.OR.A5JT | 1.34 | 0 | -16.64 | low |
| TCGA.OR.A5JV | 4.22 | 0 | -16.32 | low |
| TCGA.OR.A5JW | 5.27 | 0 | -16.40 | low |
| TCGA.OR.A5JX | 2.60 | 0 | -16.04 | high |
| TCGA.OR.A5JZ | 0.58 | 0 | -16.75 | low |
| TCGA.OR.A5K0 | 2.82 | 0 | -15.71 | high |
| TCGA.OR.A5K1 | 7.46 | 0 | -16.87 | low |
| TCGA.OR.A5K3 | 7.79 | 0 | -16.91 | low |
| TCGA.OR.A5K4 | 1.45 | 0 | -16.48 | low |
| TCGA.OR.A5K5 | 0.69 | 0 | -15.61 | high |
| TCGA.OR.A5K6 | 3.10 | 0 | -16.54 | low |
| TCGA.OR.A5K8 | 1.38 | 0 | -16.26 | high |
| TCGA.OR.A5KO | 3.87 | 0 | -16.44 | low |
| TCGA.OR.A5KT | 7.32 | 0 | -16.03 | high |
| TCGA.OR.A5KU | 12.80 | 0 | -16.10 | high |
| TCGA.OR.A5KV | 10.02 | 0 | -16.90 | low |
| TCGA.OR.A5KW | 4.18 | 0 | -16.27 | high |
| TCGA.OR.A5KX | 2.99 | 0 | -15.47 | high |
| TCGA.OR.A5L3 | 10.68 | 0 | -16.55 | low |
| TCGA.OR.A5L4 | 1.98 | 0 | -16.97 | low |
| TCGA.OR.A5L5 | 2.30 | 0 | -16.90 | low |
| TCGA.OR.A5L6 | 1.72 | 0 | -16.51 | low |
| TCGA.OR.A5L8 | 1.52 | 0 | -16.60 | low |
| TCGA.OR.A5L9 | 1.77 | 0 | -17.20 | low |
| TCGA.OR.A5LA | 1.33 | 0 | -16.98 | low |
| TCGA.OR.A5LB | 3.30 | 1 | -15.08 | high |
| TCGA.OR.A5LC | 0.44 | 1 | -12.78 | high |
| TCGA.OR.A5LD | 3.28 | 1 | -14.87 | high |
| TCGA.OR.A5LE | 1.81 | 1 | -14.42 | high |
| TCGA.OR.A5LG | 4.35 | 0 | -15.80 | high |
| TCGA.OR.A5LH | 6.53 | 1 | -16.63 | low |
| TCGA.OR.A5LJ | 3.03 | 1 | -14.54 | high |
| TCGA.OR.A5LK | 6.09 | 0 | -17.05 | low |
| TCGA.OR.A5LL | 4.42 | 1 | -15.28 | high |
| TCGA.OR.A5LM | 5.09 | 0 | -16.77 | low |
| TCGA.OR.A5LN | 5.25 | 0 | -16.88 | low |
| TCGA.OR.A5LO | 5.34 | 0 | -15.91 | high |
| TCGA.OR.A5LP | 4.34 | 0 | -16.63 | low |
| TCGA.OR.A5LR | 1.75 | 0 | -16.95 | low |
| TCGA.OR.A5LS | 2.42 | 0 | -16.29 | low |
| TCGA.OR.A5LT | 1.00 | 0 | -16.53 | low |
| TCGA.OU.A5PI | 1.94 | 0 | -15.93 | high |
| TCGA.P6.A5OF | 0.57 | 1 | -14.89 | high |
| TCGA.P6.A5OG | 1.05 | 1 | -13.53 | high |
| TCGA.PA.A5YG | 1.29 | 0 | -16.96 | low |
| TCGA.PK.A5H8 | 8.88 | 0 | -16.49 | low |
| TCGA.PK.A5H9 | 0.84 | 0 | -16.27 | high |
| TCGA.PK.A5HA | 2.27 | 0 | -15.59 | high |
| TCGA.PK.A5HB | 3.54 | 0 | -15.70 | high |

Sup Table 2. Riskscore for training set. OS, overall survival. Event: Survival status. 1 represent patient was dead. 0 represent patient was alive. The patients were classified into low‐risk group and high‐risk group according to the median value of the risk scores.

|  | OS | Event | riskScore | risk |
| --- | --- | --- | --- | --- |
| TCGA.OR.A5J3 | 1942 | 0 | -16.5226836 | low |
| TCGA.OR.A5J6 | 2428 | 0 | -16.71350926 | low |
| TCGA.OR.A5JD | 2782 | 0 | -16.09657541 | low |
| TCGA.OR.A5JF | 1259 | 0 | -15.9430128 | low |
| TCGA.OR.A5JG | 541 | 1 | -15.77993691 | high |
| TCGA.OR.A5JI | 1424 | 0 | -17.00326912 | low |
| TCGA.OR.A5JM | 562 | 1 | -14.93737515 | high |
| TCGA.OR.A5JY | 552 | 1 | -16.41991597 | low |
| TCGA.OR.A5K2 | 994 | 1 | -15.50014218 | high |
| TCGA.OR.A5K9 | 344 | 1 | -15.32397381 | high |
| TCGA.OR.A5KY | 391 | 1 | -15.39951255 | high |
| TCGA.OR.A5KZ | 125 | 1 | -14.19967597 | high |

Sup Table 3. Riskscore for testing set. OS, overall survival. Event: Survival status. 1 represent patient was dead. 0 represent patient was alive. The patients were classified into low‐risk group and high‐risk group according to the median value of the risk scores.
